# Perception Science in the Age of Deep Neural Networks

**DOI:** 10.3389/fpsyg.2017.00142

**Published:** 2017-02-02

**Authors:** Rufin VanRullen

**Affiliations:** ^1^Centre National de la Recherche Scientifique, UMR 5549, Faculté de Médecine PurpanToulouse, France; ^2^Université de Toulouse, Centre de Recherche Cerveau et Cognition, Université Paul SabatierToulouse, France

**Keywords:** perception, neuroscience, psychology, neural networks, deep learning, artificial intelligence

For decades, perception was considered a unique ability of biological systems, little understood in its inner workings, and virtually impossible to match in artificial systems. But this status quo was upturned in recent years, with dramatic improvements in computer models of perception brought about by “deep learning” approaches. What does all the ruckus about a “new dawn of artificial intelligence” imply for the neuroscientific and psychological study of perception? Is it a threat, an opportunity, or maybe a little of both?

## While we were sleeping…

My personal journey in the field of perception science started about 20 years ago. For as long as I can remember, we perception scientists have exploited in our papers and grant proposals the lack of human-level artificial perception systems, both as a justification for scientific inquiry, and as a convenient excuse for using a cautious, methodical approach—i.e., “baby steps.” Visual object recognition, for example, seemed such an intractable problem that it was obviously more reasonable to study simple stimuli (e.g., Gabor patches), or to focus on highly specific sub-components of object recognition (e.g., symmetry invariance). But now neural networks, loosely inspired by the hierarchical architecture of the primate visual system, routinely outperform humans in object recognition tasks (Krizhevsky et al., 2012; Sermanet et al., 2013; Simonyan and Zisserman, 2014; He et al., 2015; Szegedy et al., 2015). Our excuse is gone—and yet we are still nowhere near a complete description and understanding of biological vision.

It would take a monastic life over the last 5 years to be fully unaware of the recent developments in machine learning and artificial intelligence. Things that robots could only do in science fiction movies can now be performed by our smartphones, sometimes without our even noticing. We talk to Siri, Cortana, Google Assistant, or Alexa; they understand, obey, and respond with naturalistic speech and an occasional joke. Any language can be comprehended and translated near-instantaneously (Johnson et al., 2016; van den Oord et al., 2016). The same methods that have been used to crack Natural Language Processing (NLP) have also been applied to the creation of novel music (Hadjeres and Pachet, 2016; van den Oord et al., 2016) (youtube.com/watch?v=LSHZ_b05W7o or youtu.be/QiBM7-5hA6o), or to writing new texts, from novels to TV show scripts to fake (but eerily credible) Donald Trump tweets (twitter.com/deepdrumpf). Chatbots based on these algorithms are set to replace humans in many online services.

**Figure 1:**
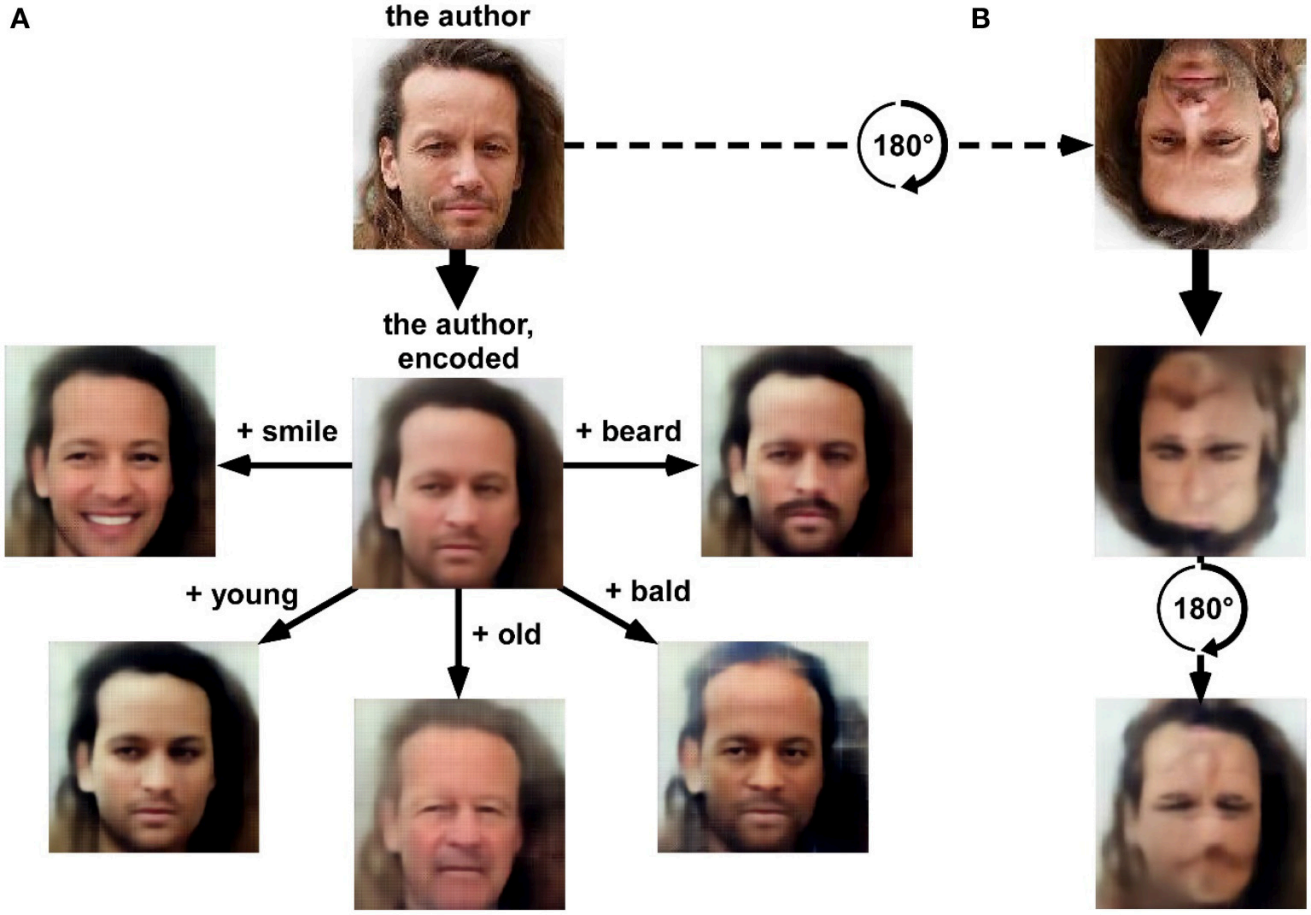
A “variational auto-encoder” (VAE) deep network (13 layers) was trained using an unsupervised “generative adversarial network” procedure (VAE/GAN, Goodfellow et al., 2014; Larsen et al., 2015) on a labeled database of 202,599 celebrity faces (15 epochs). The latent space (1024-dimensional) of the resulting network provides a description of numerous facial features that could approximate face representations in the human brain. **(A)** A picture of the author as seen (i.e., “encoded”) by the network is rendered (i.e., “decoded”) in the center of the panel. After encoding, the latent space can be sampled with simple linear algebra. For example, adding a “beard vector” (obtained by subtracting the average latent description of 1000 faces having a “no-beard” label from the average latent description of 1000 faces having a “beard” label) before decoding creates a realistic image of the author with a beard. The same operation can be done (clockwise, from right) by adding average vectors reflecting the labels “bald,” “old,” “young,” or “smile.” In short, the network manipulates concepts, which it can extract from and render to pixel-based representations. It is tempting to envision that the 1024 “hidden neurons” forming this latent space could display a pattern of stimulus selectivity comparable to that observed in certain human face-selective regions (Kanwisher et al., 1997; Tsao et al., 2006; Freiwald et al., 2009; Freiwald and Tsao, 2010). **(B)** Since the network (much like the human brain) was trained solely with upright faces, it inappropriately encodes an upside-down face, partly erasing important facial features (the mouth) and “hallucinating” inexistent features (a faint nose and mouth in the forehead region). This illustrates how human-like perceptual behavior (here, the face inversion effect) can emerge from computational principles. The database used for training this network is accessible from mmlab.ie.cuhk.edu.hk/projects/CelebA.html (Liu et al., 2015).

The staggering “creativity” of machines is also expressed in the field of image processing and machine vision. Human-level object recognition networks trained by “deep learning” were only the beginning. Now complex scenes can be analyzed to precisely localize and identify each object and its relation to others, and to provide a natural text description, e.g., “two children are playing ball on the beach” (Karpathy and Fei-Fei, 2015; Vinyals et al., 2016). By inverting the analysis process (“deconvolution”), novel images can be synthesized, giving such networks the ability to “dream” (Mordvintsev et al., 2015), but also to perform useful image processing feats. You can take a portrait and make the person smile, or look younger (Figure 1). You can give a holiday picture and have it painted like a Renoir (Gatys et al., 2015; Dumoulin et al., 2016). You can input an old black-and-white photo and have it colorized (Isola et al., 2016; Zhang et al., 2016). You can give a 3-color doodle (“here goes the lake, here are some trees, and there is the sky”) and have a realistic photo synthesized (Champandard, 2016; Isola et al., 2016). You can give a line drawing and turn it into a real object (Isola et al., 2016). You can give a low-resolution picture and have its original resolution restored (Dong et al., 2015; Romano et al., 2016). You can give a text description, and have a novel, never-seen before picture generated from scratch (Mansimov et al., 2015; Nguyen et al., 2016). There does not seem to be any limit to what can be done, except for human imagination (and training datasets).

Meanwhile, the field of Perception Science still struggles to explain how sensory information is turned into meaningful concepts by the human (or animal) brain, let alone understanding imagination, or artistic creativity. This, then, is the rather pessimistic take on the impact of this machine learning revolution for Perception Science: It forces us to take a good, hard look at our slow progress. While we were arguing over the details, somebody figured out the big picture.

## What dreams may come

But there are, of course, arguments against such a dark depiction. For one thing, machine learning still has a long way to go. There are many areas of perception science where deep neural networks (DNNs) haven't been applied yet, or have not yet met the anticipated success: For example, motion processing, ocular disparity and depth processing, color constancy, grouping and Gestalt laws, attention, perceptual multi-stability, or multi-sensory integration, just to name a few. On the other hand, it can be mathematically demonstrated that whenever there exists a reasonable solution to map inputs onto outputs, deep learning has the ability to find it. And by definition, for any perceptual science problem there is at least one reasonable solution: The one implemented in our brains. So these apparent limitations of deep learning are unlikely to hold for very long: They will be easily cracked, as soon as scientists harness sufficient motivation (which often hinges on the prospect of commercial applications), can properly assess the relevant input and output spaces, and can gather enough training data.

Moreover, there are concerns about the biological plausibility of current machine learning approaches. If our brains' abilities are emulated by algorithms that could not possibly exist in the human brain, then these artificial networks, however powerful, cannot really inform us about the brain's behavior. Such concerns include the great reliance of deep neural networks on supervised learning methods using large datasets of labeled exemplars. In contrast, humans can often learn without explicit supervision or “labels.” Unsupervised learning methods do exist for artificial neural networks, but they often give rise to a feature space that is insufficiently powerful and needs to be complemented by supervised fine-tuning in order to allow, for example, for accurate object recognition (Hinton et al., 1995, 2006; Hinton and Salakhutdinov, 2006). The large amounts of labeled training data required for deep learning can themselves be viewed as implausible. Most important perhaps is the inexistence of a generally accepted equivalent solution to the back-propagation algorithm in biological brains: This algorithm is the cornerstone of deep learning (LeCun et al., 2015), which allows gradient-descent optimization of connection weights to be performed iteratively (via the so-called “chain rule”) through the multiple layers of a network. Furthermore, there are crucial aspects of biological neural networks that are plainly disregarded in the major deep learning approaches. In particular, most state-of-the-art deep neural networks do not use spikes, and thus have no real temporal dynamics to speak of (just arbitrary, discrete time steps). This simplification implies that such networks cannot help us in understanding dynamic aspects of brain function, such as neural synchronization and oscillatory communication. Finally, the most successful deep networks so far have strongly relied on feed-forward architectures, whereas the brain includes massive feedback connections. The popular recurrent neural networks (RNN) are an exception (Hochreiter and Schmidhuber, 1997; Pascanu et al., 2013), but even they have specific short-range feedback loops that do not compare with the brain's long-range connectivity (and the existence of communication “hubs,” like the thalamus).

All these deviations from known biological properties, often motivated by considerations of computational efficiency, do not constitute real barriers, and recent work is starting to reconcile machine learning and brain reality on most of these fronts. Unsupervised and semi-supervised learning methods have been suggested that require no or only few occasional labels to be provided (Anselmi et al., 2013; Doersch et al., 2015; Wang and Gupta, 2015). Some of these methods can also learn features and representations from one or just a few exemplars, a form of “one-shot learning” on par with human capabilities (Anselmi et al., 2013; Rezende et al., 2016; Santoro et al., 2016). At least certain forms of backpropagation appear compatible with a number of biological observations, e.g., spike timing-dependent plasticity (Scellier and Bengio, 2016). Deep neural networks that use spikes are becoming commonplace (Yu et al., 2013; Cao et al., 2015; Diehl et al., 2015; Hunsberger and Eliasmith, 2016; Kheradpisheh et al., 2016a; Lee et al., 2016; Zambrano and Bohte, 2016), and attempts have also been made to introduce oscillatory components in deep networks (Rao and Cecchi, 2011, 2013; Reichert and Serre, 2013). Finally, new DNN architectures are emerging with long-range feedforward (Huang et al., 2016a,b) and feedback connectivity (Pascanu et al., 2013; Zilly et al., 2016). In summary, it would be shortsighted to discard deep learning as irrelevant for understanding biological perception, simply based on its currently imperfect biological plausibility.

A possibly deeper limitation of machine learning lies in the argument that merely replicating behavior in an artificial system does not imply any understanding of the underlying function. In this view, we perception scientists are still left with all the work to do for the latter. But now, we are not limited anymore to studying biological systems through measurements of external behavior or through sparse and nearly-random samplings of neural activity—we can also scrutinize their artificial cousins, the deep neural networks, for which every neuron's activation function is readily accessible, and in which systematic investigations can thus prove much easier.

## A wake-up call for perception science

Thankfully, there are many other reasons to view the recent machine learning advances in an optimistic light. It is likely that the image and sound synthesis abilities of deep networks (e.g., Figure 1) will serve in the near future as a significant source of well-controlled experimental stimuli, and innovative new experimental designs. Gradient descent can be applied, for example, to create images of objects that will be recognized by humans but not by state-of-the-art deep networks (by designing a “loss function” ensuring that image content is preserved in early layers of the network, but abolished in the final layers), or conversely, non-sense images that “fool” a deep network into recognizing a given object (by inverting the aforementioned loss function) (Nguyen et al., 2014). Which brain regions would respond to the latter, and which to the former? How would event-related potentials, or brain oscillatory activity, react to each image type? Could certain “selective” behaviors (e.g., rapid selective eye movements) be preserved in the absence of explicit recognition?

Deep learning can also turn out to be a source of powerful new data analysis tools. Neuroscience and psychological experiments produce masses of data that can prove challenging for conventional analysis methods. Some 10 or 12 years ago, multivariate pattern analysis (MVPA) methods promised to open new avenues for neuroscience research (Haynes and Rees, 2005; Kamitani and Tong, 2005). Similarly, deep networks could now become a key to reveal the complex mapping between sensory inputs, brain signals and behavioral outputs, and unlock the mysteries of the brain.

Moreover, deep neural networks are also suited to serve a more indirect role in Perception Science, not as a methods tool but as a source of inspiration for existing and novel theories about brain function. Many studies have already started to characterize the existing relations (and differences) between patterns of activity obtained from specific layers of deep networks, and from specific brain regions (Cadieu et al., 2014; Khaligh-Razavi and Kriegeskorte, 2014; Güçlü and van Gerven, 2015; Cichy et al., 2016a,b) or from human behavior (Kheradpisheh et al., 2016b,c). As alluded to in Figure 1B, the powerful latent representation spaces generated by deep neural networks could be used, for example, to study the face inversion effect. They could also help address the debate between expertise vs. domain-specificity in face processing (Kanwisher et al., 1997; Gauthier et al., 1999, 2000; Tarr and Gauthier, 2000; Rossion et al., 2004; Tsao et al., 2006; Freiwald et al., 2009; Freiwald and Tsao, 2010), or between modular vs. distributed object representations (Haxby et al., 2001; Reddy and Kanwisher, 2006), and possibly many others.

Finally, and perhaps most importantly, we should view the amazing recent progress of machine learning as a wake-up call, an occasion to abandon our excuses, and a reason to embolden our approaches. No more “baby steps” for us—the time is ripe to address the big picture.

## Forward-looking statement

How does our journal fit in this global context? As usual, Frontiers in Perception Science will continue to welcome all original research papers that explore perception in and across any modalities, whether in animals, humans or—why not?—machines, using methods drawn from neuroscience and psychology (but also mathematics, engineering, and computer science). The main criterion for publication is scientific rigor and soundness applied to the study's motivations, methods, and interpretation. Perceived impact or newsworthiness are not relevant factors. While plagiarism is evidently prohibited, explicit replications of previous studies will be viewed favorably. Importantly, these (and any other) papers can equally report positive or negative outcomes –as long as the methodology is rigorous. We hope that we can thereby contribute to resorbing the current confidence crisis in neuroscience and psychology (Ioannidis, 2005; Simmons et al., 2011; Open Science, 2015; Gilbert et al., 2016). Finally, the journal publishes a number of article formats that are complementary to original research and constitute an important resource for the field, such as methods articles, reviews or mini-reviews, perspectives, opinions, and commentaries, hypothesis & theory papers. For these publications as well, the main criterion remains scientific rigor and soundness.

To conclude, as the above arguments should make clear, I believe that the success of deep learning at emulating biological perception is a game-changer that our field cannot ignore. It would be like lighting a fire by hitting stones, with a flamethrower lying on our side. On the other hand, while I formulate the convergence between biological and machine perception (Cox and Dean, 2014; Kriegeskorte, 2015; Marblestone et al., 2016) as both a wish and a prediction for the future of Perception Science as a whole, it is evident that many individual papers or researchers in the field will not be systematically concerned with deep learning. That's still okay—if that is your case, Frontiers in Perception Science will remain a venue of choice for your paper. Just don't motivate it by the “inability of machine perception to achieve human-level performance”: That would be shortsighted.

## Author contributions

The author confirms being the sole contributor of this work and approved it for publication.

### Conflict of interest statement

The author declares that the research was conducted in the absence of any commercial or financial relationships that could be construed as a potential conflict of interest.

## References

[B1] Anselmi F. Leibo J. Z. Rosasco L. Mutch J. Tacchetti A. Poggio T. A. (2013). Unsupervised learning of invariant representations in hierarchical architectures. CoRR 1311.4158.

[B2] Cadieu C. F. Hong H. Yamins D. L. Pinto N. Ardila D. Solomon E. A.. (2014). Deep neural networks rival the representation of primate IT cortex for core visual object recognition. PLoS Comput. Biol. 10:e1003963. 10.1371/journal.pcbi.1003963 25521294 PMC4270441

[B3] Cao Y. Chen Y. Khosla D. (2015). Spiking deep convolutional neural networks for energy-efficient object recognition. Int. J. Comp. Vis. 113, 54–66. 10.1007/s11263-014-0788-3

[B4] Champandard A. J. (2016). Semantic style transfer and turning two-bit doodles into fine artworks. CoRR 1603.01768.

[B5] Cichy R. M. Khosla A. Pantazis D. Oliva A. (2016a). Dynamics of scene representations in the human brain revealed by magnetoencephalography and deep neural networks. Neuroimage. [Epub ahead of print]. 10.1016/j.neuroimage.2016.03.063 27039703 PMC5542416

[B6] Cichy R. M. Khosla A. Pantazis D. Torralba A. Oliva A. (2016b). Comparison of deep neural networks to spatio-temporal cortical dynamics of human visual object recognition reveals hierarchical correspondence. Sci. Rep. 6:27755. 10.1038/srep27755 27282108 PMC4901271

[B7] Cox D. D. Dean T. (2014). Neural networks and neuroscience-inspired computer vision. Curr. Biol. 24, R921–R929. 10.1016/j.cub.2014.08.026 25247371

[B8] Diehl P. U. Neil D. Binas J. Cook M. Liu S. C. Pfeiffer M. (2015). Fast-classifying, high-accuracy spiking deep networks through weight and threshold balancing 2015, in International Joint Conference on Neural Networks (IJCNN) (Killarney), 1–8.

[B9] Doersch C. Gupta A. Efros A. A. (2015). Unsupervised visual representation learning by context prediction. CoRR 1505.05192.

[B10] Dong C. Loy C. C. He K. Tang X. (2015). Image super-resolution using deep convolutional networks. CoRR 1501.00092. 26761735 10.1109/TPAMI.2015.2439281

[B11] Dumoulin V. Shlens J. Kudlur M. (2016). A learned representation for artistic style. CoRR 1610.07629.

[B12] Freiwald W. A. Tsao D. Y. (2010). Functional compartmentalization and viewpoint generalization within the macaque face-processing system. Science 330, 845–851. 10.1126/science.1194908 21051642 PMC3181095

[B13] Freiwald W. A. Tsao D. Y. Livingstone M. S. (2009). A face feature space in the macaque temporal lobe. Nat. Neurosci. 12, 1187–1196. 10.1038/nn.2363 19668199 PMC2819705

[B14] Gatys L. A. Ecker A. S. Bethge M. (2015). A neural algorithm of artistic style. CoRR 1508.06576.

[B15] Gauthier I. Skudlarski P. Gore J. C. Anderson A. W. (2000). Expertise for cars and birds recruits brain areas involved in face recognition. Nat. Neurosci. 3, 191–197. 10.1038/72140 10649576

[B16] Gauthier I. Tarr M. J. Anderson A. W. Skudlarski P. Gore J. C. (1999). Activation of the middle fusiform ‘face area’ increases with expertise in recognizing novel objects. Nat. Neurosci. 2, 568–573. 10.1038/9224 10448223

[B17] Gilbert D. T. King G. Pettigrew S. Wilson T. D. (2016). Comment on “Estimating the reproducibility of psychological science”. Science 351, 1037 10.1126/science.aad7243 26941311

[B18] Goodfellow I. J. Pouget-Abadie J. Mirza M. Xu B. Warde-Farley D. Ozair S. (2014). Generative adversarial networks. ArXiv e-prints arXiv: 1406.2661.

[B19] Güçlü U. van Gerven M. A. (2015). Deep neural networks reveal a gradient in the complexity of neural representations across the ventral stream. J. Neurosci. 35, 10005–10014. 10.1523/JNEUROSCI.5023-14.2015 26157000 PMC6605414

[B20] Hadjeres G. Pachet F. (2016). DeepBach: a Steerable Model for Bach chorales generation. arXiv preprint arXiv: 1612.01010.

[B21] Haxby J. V Gobbini M. I Furey M. L. Ishai A. Schouten J. L. Pietrini P. (2001). Distributed and overlapping representations of faces and objects in ventral temporal cortex. Science 293, 2425–2430. 10.1126/science.1063736 11577229

[B22] Haynes J. D. Rees G. (2005). Predicting the orientation of invisible stimuli from activity in human primary visual cortex. Nat. Neurosci. 8, 686–691. 10.1038/nn1445 15852013

[B23] He K. Zhang X. Ren S. Sun J. (2015). Deep residual learning for image recognition. CoRR 1512.03385.

[B24] Hinton G. E. Dayan P. Frey B. J. Neal R. M. (1995). The “wake-sleep” algorithm for unsupervised neural networks. Science 268, 1158–1161. 7761831 10.1126/science.7761831

[B25] Hinton G. E. Osindero S. Teh Y. W. (2006). A fast learning algorithm for deep belief nets. Neural Comput. 18, 1527–1554. 10.1162/neco.2006.18.7.1527 16764513

[B26] Hinton G. E. Salakhutdinov R. R. (2006). Reducing the dimensionality of data with neural networks. Science 313, 504–507. 10.1126/science.1127647 16873662

[B27] Hochreiter S. Schmidhuber J. (1997). Long short-term memory. Neural Comput. 9, 1735–1780. 10.1162/neco.1997.9.8.1735 9377276

[B28] Huang G. Liu Z. Weinberger K. Q. (2016a). Densely connected convolutional networks. CoRR 1608.06993.

[B29] Huang G. Sun Y. Liu Z. Sedra D. Weinberger K. Q. (2016b). Deep networks with stochastic depth. CoRR 1603.09382.

[B30] Hunsberger E. Eliasmith C. (2016). Training spiking deep networks for neuromorphic hardware. CoRR 1611.05141.

[B31] Ioannidis J. P. (2005). Why most published research findings are false. PLoS Med. 2:e124. 10.1371/journal.pmed.0020124 16060722 PMC1182327

[B32] Isola P. Zhu J.-Y. Zhou T. Efros A. A. (2016). Image-to-image translation with conditional adversarial networks. CoRR 1611.07004.

[B33] Johnson M. Schuster M. Le Q. V Krikun M. Wu Y. Chen Z. (2016). Google's multilingual neural machine translation system: enabling zero-shot translation. CoRR 1611.04558.

[B34] Kamitani Y. Tong F. (2005). Decoding the visual and subjective contents of the human brain. Nat. Neurosci. 8, 679–685. 10.1038/nn1444 15852014 PMC1808230

[B35] Kanwisher N. McDermott J. Chun M. M. (1997). The fusiform face area: a module in human extrastriate cortex specialized for face perception. J. Neurosci. 17, 4302–4311. 9151747 10.1523/JNEUROSCI.17-11-04302.1997 PMC6573547

[B36] Karpathy A. Fei-Fei L. (2015). Deep visual-semantic alignments for generating image descriptions, in Proceedings of the IEEE Conference on Computer Vision and Pattern Recognition (Boston, MA), 3128–3137. 10.1109/TPAMI.2016.2598339 27514036

[B37] Khaligh-Razavi S. M. Kriegeskorte N. (2014). Deep supervised, but not unsupervised, models may explain IT cortical representation. PLoS Comput. Biol. 10:e1003915. 10.1371/journal.pcbi.1003915 25375136 PMC4222664

[B38] Kheradpisheh S. R. Ganjtabesh M. Thorpe S. J. Masquelier T. (2016a). STDP-based spiking deep neural networks for object recognition. CoRR 1611.01421. 10.1016/j.neunet.2017.12.005 29328958

[B39] Kheradpisheh S. R. Ghodrati M. Ganjtabesh M. Masquelier T. (2016b). Deep networks can resemble human feed-forward vision in invariant object recognition. Sci. Rep. 6:32672. 10.1038/srep32672 27601096 PMC5013454

[B40] Kheradpisheh S. R. Ghodrati M. Ganjtabesh M. Masquelier T. (2016c). Humans and deep networks largely agree on which kinds of variation make object recognition harder. Front. Comput. Neurosci. 10:92. 10.3389/fncom.2016.00092 27642281 PMC5015476

[B41] Kriegeskorte N. (2015). Deep neural networks: a new framework for modeling biological vision and brain information processing. Annu. Rev. Vis. Sci. 1, 417–446. 10.1146/annurev-vision-082114-035447 28532370

[B42] Krizhevsky A. Sutskever I. Hinton G. E. (2012). ImageNet classification with deep convolutional neural networks, in Advances in Neural Information Processing Systems 25 (Lake Tahoe, NV), 1097–1105.

[B43] Larsen A. B. L. Sønderby S. K. Winther O. (2015). Autoencoding beyond pixels using a learned similarity metric. CoRR 1512.09300.

[B44] LeCun Y. Bengio Y. Hinton G. (2015). Deep learning. Nature 521, 436–444. 10.1038/nature14539 26017442

[B45] Lee J. H. Delbruck T. Pfeiffer M. (2016). Training deep spiking neural networks using backpropagation. Front. Neurosci. 10:508. 10.3389/fnins.2016.00508 27877107 PMC5099523

[B46] Liu Z. Luo P. Wang X. Tang X. (2015). Deep learning face attributes in the wild, in Proceedings of International Conference on Computer Vision (Santiago: ICCV).

[B47] Mansimov E. Parisotto E. Ba L. J. Salakhutdinov R. (2015). Generating images from captions with attention. CoRR 1511.02793.

[B48] Marblestone A. H. Wayne G. Kording K. P. (2016). Toward an integration of deep learning and neuroscience. Front. Comput. Neurosci. 10:94. 10.3389/fncom.2016.00094 27683554 PMC5021692

[B49] Mordvintsev A. Olah C. Tyka M. (2015). Inceptionism: Going Deeper into Neural Networks. Google Research Blog.

[B50] Nguyen A. M. Yosinski J. Clune J. (2014). Deep neural networks are easily fooled: high confidence predictions for unrecognizable images. CoRR 1412.1897.

[B51] Nguyen A. Yosinski J. Bengio Y. Dosovitskiy A. Clune J. (2016). Plug & play generative networks: conditional iterative generation of images in latent space. arXiv preprint arXiv: 1612.00005.

[B52] Open Science C. (2015). PSYCHOLOGY. Estimating the reproducibility of psychological science. Science 34:aac4716 10.1126/science.aac4716 26315443

[B53] Pascanu R. Gülcehre C Cho K. Bengio Y. (2013). How to construct deep recurrent neural networks. CoRR 1312.6026.

[B54] Rao A. R. Cecchi G. (2013). Capacity limits in oscillatory networks: Implications for sensory coding, in The 2013 International Joint Conference on Neural Networks (IJCNN) (Dallas, TX), 1–8.

[B55] Rao A. R. Cecchi G. A. (2011). The effects of feedback and lateral connections on perceptual processing: A study using oscillatory networks Neural Networks (IJCNN), in The 2011 International Joint Conference on Neural Networks (San Jose, CA), 1177–1184.

[B56] Reddy L. Kanwisher N. (2006). Coding of visual objects in the ventral stream. Curr. Opin. Neurobiol. 16, 408–414. 10.1016/j.conb.2006.06.004 16828279

[B57] Reichert D. P. Serre T. (2013). Neuronal synchrony in complex-valued deep networks. arXiv preprint arXiv: 1312.6115.

[B58] Rezende D. J. Mohamed S. Danihelka I. Gregor K. Wierstra D. (2016). One-shot generalization in deep generative models. arXiv preprint arXiv: 1603.05106.

[B59] Romano Y. Isidoro J. Milanfar P. (2016). RAISR: rapid and accurate image super resolution. CoRR 1606.01299.

[B60] Rossion B. Kung C. C. Tarr M. J. (2004). Visual expertise with nonface objects leads to competition with the early perceptual processing of faces in the human occipitotemporal cortex. Proc. Natl. Acad. Sci. U.S.A. 101, 14521–14526. 10.1073/pnas.0405613101 15448209 PMC521961

[B61] Santoro A. Bartunov S. Botvinick M. Wierstra D. Lillicrap T. P. (2016). One-shot learning with memory-augmented neural networks. CoRR 1605.06065.

[B62] Scellier B. Bengio Y. (2016). Towards a biologically plausible backprop. arXiv preprint arXiv: 1602.05179.

[B63] Sermanet P. Eigen D. Zhang X. Mathieu M. Fergus R. LeCun Y. (2013). OverFeat: integrated recognition, localization and detection using convolutional networks. CoRR 1312.6229.

[B64] Simmons J. P. Nelson L. D. Simonsohn U. (2011). False-positive psychology: undisclosed flexibility in data collection and analysis allows presenting anything as significant. Psychol. Sci. 22, 1359–1366. 10.1177/0956797611417632 22006061

[B65] Simonyan K. Zisserman A. (2014). Very deep convolutional networks for large-scale image recognition. CoRR 1409.1556.

[B66] Szegedy C. Liu W. Jia Y. Sermanet P. Reed S. Anguelov D. (2015). Going deeper with convolutions, in Proceedings of the IEEE Conference on Computer Vision and Pattern Recognition (Boston, MA), 1–9.

[B67] Tarr M. J. Gauthier I. (2000). FFA: a flexible fusiform area for subordinate-level visual processing automatized by expertise. Nat. Neurosci. 3, 764–769. 10.1038/77666 10903568

[B68] Tsao D. Y. Freiwald W. A. Tootell R. B. Livingstone M. S. (2006). A cortical region consisting entirely of face-selective cells. Science 311, 670–674. 10.1126/science.1119983 16456083 PMC2678572

[B69] van den Oord A. Dieleman S. Zen H. Simonyan K. Vinyals O. Graves A. (2016). WaveNet: a generative model for raw audio. CoRR 1609.03499.

[B70] Vinyals O. Toshev A. Bengio S. Erhan D. (2016). Show and tell: lessons learned from the 2015 MSCOCO image captioning challenge. IEEE Trans. Pattern Anal. Mach. Intell. 1 10.1109/TPAMI.2016.2587640 28055847

[B71] Wang X. Gupta A. (2015). Unsupervised learning of visual representations using videos. CoRR 1505.00687.

[B72] Yu Q. Tang H. Tan K. C. Li H. (2013). Rapid feedforward computation by temporal encoding and learning with spiking neurons. IEEE Trans. Neural Netw. Learn Syst. 24, 1539–1552. 10.1109/TNNLS.2013.2245677 24808592

[B73] Zambrano D. Bohte S. M. (2016). Fast and efficient asynchronous neural computation with adapting spiking neural networks. CoRR 1609.02053.

[B74] Zhang R. Isola P. Efros A. A. (2016). Colorful image colorization. CoRR 1603.08511.

[B75] Zilly J. G. Srivastava R. K. Koutník J. Schmidhuber J. (2016). Recurrent highway networks. CoRR 1607.03474.

